# Lesion Location-Based Prediction of Visual Field Improvement after Cerebral Infarction

**DOI:** 10.1371/journal.pone.0143882

**Published:** 2015-11-25

**Authors:** Bum Joon Kim, Yong-Hwan Kim, Namkug Kim, Sun U. Kwon, Sang Joon Kim, Jong S. Kim, Dong-Wha Kang

**Affiliations:** 1 Department of Neurology, Asan Medical Center, University of Ulsan College of Medicine, Seoul, Seoul Korea; 2 Vision, Image and Learning Laboratory, Asan Institute for Life Sciences, Asan Medical Center, Seoul, South Korea; 3 Department of Radiology, Asan Medical Center, University of Ulsan College of Medicine, Seoul, Seoul Korea; 4 Department of Convergence Medicine, Asan Medical Center, University of Ulsan College of Medicine, Seoul, Seoul Korea; INSERM U894, FRANCE

## Abstract

**Background:**

Although the prognosis of ischemic stroke is highly dependent on the lesion location, it has rarely been quantitatively utilized. We investigated the usefulness of regional extent of ischemic lesion (rEIL) predicting the improvement of visual field defect (VFD) in patients with posterior cerebral artery infarction.

**Methods:**

The rEILs were measured in each individual cortex after transforming the lesions to a standard atlas. Significant improvement of VFD was tentatively defined as 20% improvement at 3 months after stroke. The performances of clinical and imaging variables predicting significant improvement were measured by support vector machine. The maximum performance of variables predicting the significant improvement was compared between subgroups of variables (clinical, baseline severity and lesion volume) and the effect of adding rEIL to those subgroups of variables was evaluated.

**Results:**

A total of 35 patients were enrolled in this study. Left PCA infarct, MR-time from onset, rEILs in the lingual, calcarine, and cuneus cortices were good prognostic indicators of hemi-VFD (performance for predicting the significant improvement: 72.8±11.8%, 66.1±11.2%, respectively). A combination of the rEILs of each cortical subregions demonstrated a better predictive performance for hemi-VFD (83.8±9.5%) compared to a combination of clinical variables (72.8±11.8; p<0.001), baseline severity (63.0±11.9%; p<0.001), or lesion volume (62.6±12.7%; p<0.001). Adding a rEIL to other variables improved the prognostic prediction for hemi-VFD (74.4±11.6% to 91.3±7.7%; p<0.001).

**Conclusions:**

An estimation of rEIL provides useful information regarding the ischemic lesion location. rEIL accurately predicts the significant improvement of VFD and enhances the prediction power when combined with other variables.

## Introduction

Understanding the natural course and prognosis helps to guide the management of ischemic stroke. [1] The severity of neurological deficit, patient’s age, comorbid diseases and the mechanism of stroke are known to affect functional outcome. [2,3] Approaches to predict post-stroke recovery using these clinical variables may provide a convenient method to assess the prognosis. However, these factors predict only less than 50% of variance in stroke recovery. [4] In addition, while some clinical variables are consistently associated with stroke prognosis among previous studies, other variables show variations.

The topographical lesion location determines the type and severity of neurological deficits. The location of the ischemic lesion was associated with functional outcome [5] and more specifically, involvement of the cerebral cortex was associated with poor functional recovery. [6,7] Finally, the prognosis of neurological deficits was well predicted by atlas-based lesion coding. [8] However, still quantitative analyses of the extent of ischemic damage in each cortex for predicting the prognosis has not been widely accepted.

Unlike other impairments caused by stroke (e.g. motor or language dysfunction), the lesion location causing visual field defect (VFD) is less diverse. Usually, the lesion causing VFD is located at the area supplied by posterior cerebral artery (PCA) and the lesion location well-correlates with the severity of VFD objectively measured by perimetry. [9] Therefore, a prediction model of VFD to assess the extent of ischemic damage in an individual visual cortex may be appropriate to evaluate the usefulness of lesion location-based prediction of prognosis after ischemic stroke. Here, we tried to verify the value of assessing the regional extent of ischemic lesion (rEIL) in individual visual cortices using the initial diffusion-weighted image (DWI) for the prediction of VFD improvement after stroke.

## Materials and Methods

### Subjects and study design

Ischemic stroke patients with VFD caused by PCA infarction were prospectively enrolled between September 2011 and July 2014. Patients were included if they were older than 20 years of age and if they were admitted to Asan Medical Center within one week of symptom onset. Patients were excluded if they (1) received thrombolysis, (2) had a previous history of stroke, (3) had new vascular events (*e*.*g*., hemorrhagic transformation or recurrent ischemic stroke) between enrollment and final follow-up, and/or (4) had visual field test results that were unreliable due to being clinically inconsistent and incomplete (e.g. frequent fixation loss and false negative or positive responses)

Demographics and risk factors of each patient were prospectively obtained. All patients underwent MRI scanning at admission and two visual field tests during the study: within one week (baseline) and at 3 months of symptom onset (follow-up). The visual field score was calculated according to the results of perimetry (see below). A higher score represented a less severe VFD.

Enrolled patients were assigned into one of two groups to perform a classification test. A visual field score increase of more or less than 20% from baseline to three months was arbitrarily regarded as a significant or poor VFD improvement, respectively. This cut-off value was chosen because it was close to the median value of improvement among all patients. The Institutional Review Board of the Asan Medical Center approved this study. Written informed consent was obtained from the patients or their legally authorized representatives.

### Visual field test and analysis

VFD was quantitatively measured by a Humphrey Field Analyzer *(HFA 750i*, *program 30–2*, *SITA-Fast strategy; Zeiss-Humphrey*, *Leandro*, *CA)*. Patients were asked to respond when the short light stimuli appeared with their head fixed in position. The subjects were asked to fixate a fixation spot to reduce the influence of eye movements. From the visual field test, whole visual field consists of 76 points (19 points for each quadrant) for each eye. Each point is presented as a standard deviation from the mean score of an age-matched normal population (i.e. total deviation score). First, the average visual field score of a particular point was calculated by averaging the scores of both eyes. Then, by summating the average visual field score of each point in individual hemi-field and upper- or lower-quadrants of those who had a defect at the particular field were regarded as the visual field score of hemi- and upper- or lower-VFD, respectively. The whole visual field was categorized into four quadrants, upper-affected side (upper-VFD), upper-unaffected side, lower-affected side (lower-VFD), and lower-unaffected side. Defects on the affected side of the upper- and lower quadrants were considered as defect on hemi-field.

### Analysis and quantification of ischemic lesion

Patients underwent 1.5-T MRI scanning *(Siemens Avanto*, *Siemens Medical Solutions)*. DWIs were obtained by a single-shot spin-echo echo-planar imaging technique with repetition time of 3000 ms, echo time of 86 ms, 0.651 × 0.651 × 5 mm^3^ resolution with 2 mm gap, 384 × 384 in-plane voxels. Additionally, patients underwent 3-T MRI scanning *(Philips Achieva; Philips Medical Systems)* at 1 week after symptom onset to obtain high-resolution T1-weighted MR images (MP-RAGE, voxel size = 1 × 1 × 1 mm^3^).

An ischemic lesion mask was extracted using FSLView toolbox in FMRIB Software Library (FSL) in its native DWI space [10]. The ischemic lesion was delineated by manual drawing of the margin of the DWI lesion by an investigator (Y.-H.K) under supervision by a stroke neurologist (D.-W.K) who were blind to any clinical information. The lesion volume was calculated in its native space.

**Fig 1:**
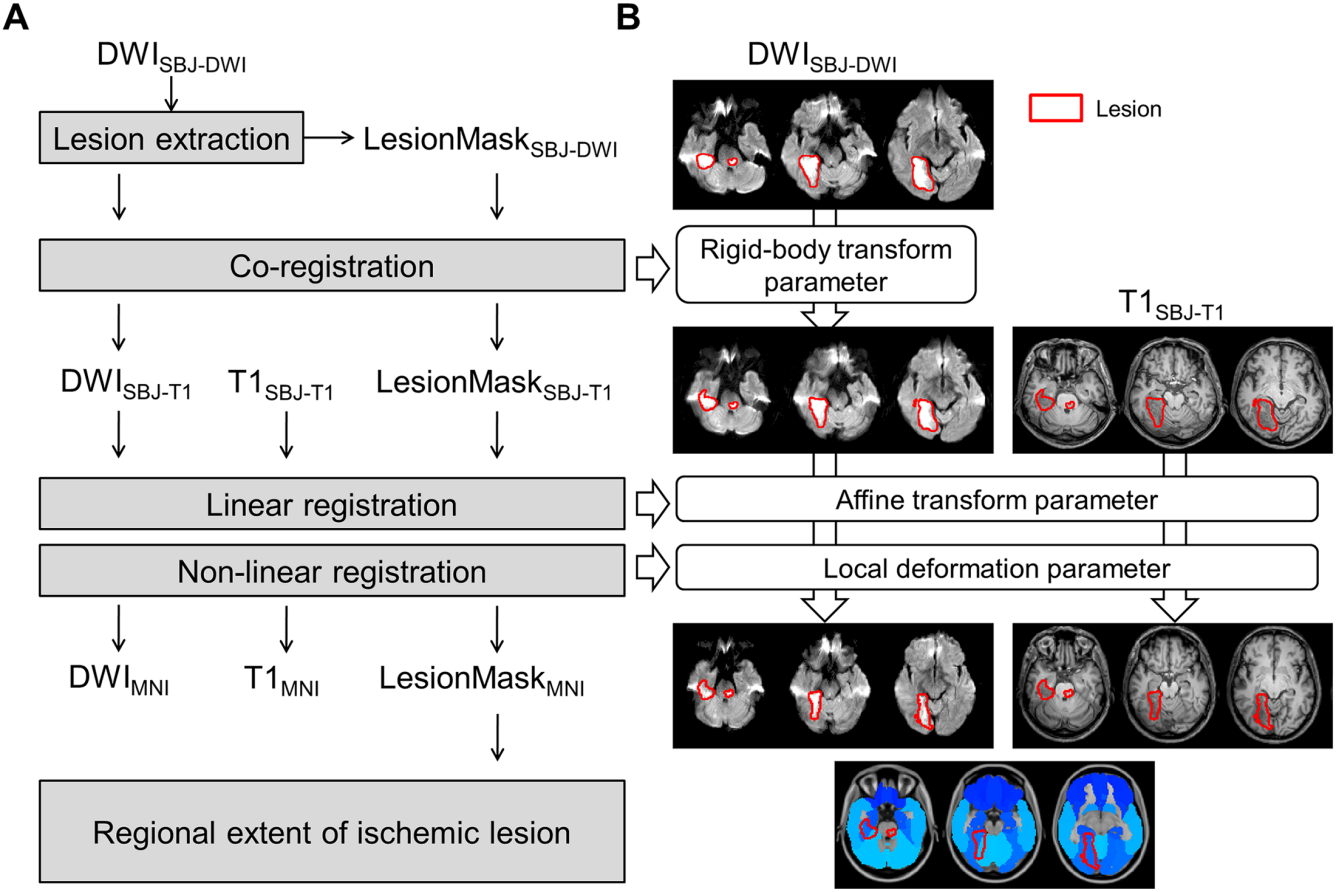
Schematic diagram of lesion quantification. (A) Process of registration into standard MNI space. (B) Exemplar images for each registration stage. Image modalities were denoted (DWI = diffusion weighted image; LesionMask = delineated binary lesion mask image; T1 = T1-weighted image). The image spaces during the registration process were denoted by sub-script (e.g., SBJ-DWI = individual subjects’ DWI space; SBJ-T1 = individual high-resolution T1 space; and MNI = standard MNI space).

Registration of the lesion mask from its native space to a standard Montreal Neurological Institute (MNI) space was performed via the FMRIB Linear or Non-linear Image Registration Tool implemented in FSL (FLIRT or FNIRT, respectively). In detail, co-registration was performed to estimate the rigid body transformation parameter between an individual apparent diffusion coefficient scan and a high-resolution individual T1 scan via FLIRT with normalized mutual information cost function. Spatial normalization was performed to estimate affine transformation and local deformation parameters between a high-resolution T1 scan and the standard MNI T1 scan via FLIRT and FNIRT with excluding lesion mask option in the non-linear registration stage. The lesion mask in diffusion space was transformed into standard MNI space by applying the rigid body transformation, affine transformation and local deformation parameters. The rEIL in each individual cortex was calculated on the automated anatomical labeling (AAL) atlas in the standard MNI space [11] to quantitatively calculate the regional damage as follows: (the volume of lesion inside the individual gyrus / the total volume of individual gyrus)×100 (%) (Fig 1).

### Statistical analysis and classification test

Clinical variables, baseline visual field score, lesion volume, and rEIL were compared between the two groups (significant vs. poor improvement groups). Chi-square test, Student’s t-test, and Fisher’s exact test were used as appropriate. The variables were also test by the correlation analysis to evaluate association with relative changes of visual field scores. A two-tailed *p* < 0.05 was considered statistically significant. To compare the voxel-wise frequency difference of lesion between the significant and poor improvement groups, Bernoulli model based two sample t-test was performed for each voxel. (12) The false discovery rate correction with significance level at *q* < 0.05 was employed with considering the multiple comparisons in the voxel-wise test.[12] All statistical analyses except for support vector machine (SVM) were performed using SPSS for Windows (*version 17*.*0; SPSS Inc*.).

For classification tests, a linear kernel-based SVM *(implemented in MATLAB with ‘svmtrain’ and ‘svmclassify’ modules*, *MathWorks*, *Inc*., *Natick*, *MA)* was adopted to train and validate the prognosis classifier. [13] Each patient was assigned into one of two classes: a significant and poor improvement group. For the test-retest of classification performance, 70% of the enrolled patients from both the significant and poor improvement groups were randomly selected and used to train the classifier. The remaining patients were used to test the performance of the classifier. Performance evaluation was conducted 100 times with randomly assigned training and test sets. Given a set of data (N × M; N = number of subjects and M = number of variables), all the possible combinations of variables (*i*.*e*., 2^M^) were considered. Best performance of a combination in a given set was determined based on the highest *t*-score from the one sample *t*-test between classification performances performed 100 times and at random chance.

First, all variables were solely tested (*i*.*e*., M = 1). Second, variable categorized as subgroups—clinical variables (M = 9), baseline visual field score (M = 1), initial lesion volume (M = 1), and rEILs (M = 9)—were tested to identify the best subgroup predicting the improvement of VFD with the highest performance. Finally, multivariate classification tests were performed by combining various subgroups of variables to identify the effect of adding rEIL to the previously known predictive factors. A paired t-test (*i*.*e*., trained classifier with same training set) was performed between the best performances of each subgroup of variables. A two tailed *p* < 0.05 with Bonferroni correction was considered statistically significant.

## Results

### Subjects and visual field improvement

During the study period, 51 patients, admitted to our stroke center with VFD and PCA infarction, were screened. Of these, 9 patients refused to participate in the study. Among the initially included 44 patients, 9 patients were further excluded (3 patients with suboptimal visual field test data due to poor compliance, another 2 patients with stroke recurrence, and 4 patients with hemorrhagic transformation during follow-up).Finally, 35 patients were enrolled.

**Fig 2:**
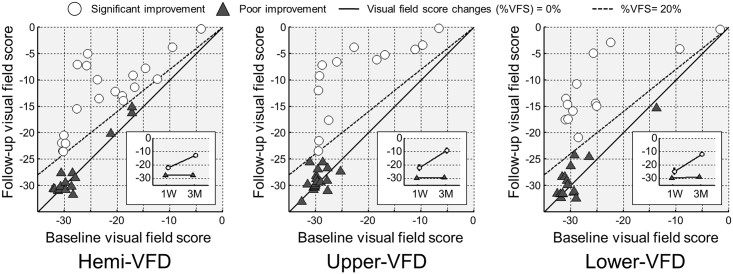
The improvement of VFD. Scatter plots show baseline VFS (i.e., at 1 week; x-axis) and follow-up VFS (i.e., at 3 months; y-axis) for hemi-VFD (left), upper-VFD (middle) and lower-VFD (right) subgroups. In each VFD subgroup, the significant improvement was defined who showed %VFS greater than 20%. The subjects with significant and poor improvement were plotted with white filled circle and with gray filled triangle, respectively. Mean and standard error plots at baseline and follow-up were shown in sub-box. VFS = average visual field score in each hemi-field, upper-quadrant or lower-quadrant, respectively; %VFS = (follow-up VFS—baseline VFS) / |baseline VFS|×100(%).

The mean age was 61.6 (SD 11.0) years, and 28 patients (80.0%) were men. Of the 35 patients, 26 (74.3%) had homonymous hemianopia, while the remaining 9 patients had quadrantanopia. By quadrants, 32 patients had upper-VFD, and 29 patients had lower-VFD. Of them, 20 of 35 patients (57.1%) with hemi-VFD, 13 of 32 patients (40.6%) with upper-VFD, and 13 of 29 patients (44.8%) with lower-VFD were classified as having a significant improvement (Fig 2). Patients with significant hemi-VFD improvement demonstrated a less severe baseline VFD, more frequent infarct location in the left hemisphere (p = 0.01) and shorter interval between stroke onset and MR time (p = 0.03) than patients with a poor hemi-VFD improvement. Significant upper-VFD improvement was also associated with higher baseline visual field score (*p* < 0.001) and initial lesion volume (*p* = 0.03) compared to that of patients with poor upper-VFD improvement. However, in terms of lower-VFD improvement, baseline visual field score or initial ischemic lesion volume was not different, whereas infarct on left PCA (*p* = 0.04), atrial fibrillation (*p* = 0.03) earlier MR time (*p* = 0.009) was associated with patients with significant lower-VFD improvement (Table 1).

**Table 1:** Comparison between patients with significant and poor VFD improvement.

	Hemi-VFD			Upper-VFD			Lower-VFD		
	Significant (n = 20)	Poor (n = 15)	*p*	Significant (n = 13)	Poor (n = 19)	*p*	Significant (n = 13)	Poor (n = 16)	*p*
**Clinical variables**									
Age (y)	63.8 [8.1]	58.7 [13.8]	0.18	64.2 [8.7]	60.6 [12.4]	0.37	64.5 [6.4]	59.2 [13.4]	0.20
Male	15 (75.0)	13 (86.7)	0.39	9 (69.2)	16 (84.2)	0.31	10 (76.9)	13 (81.2)	0.78
Left PCA infarction	14 (70.0)	4 (26.7)	0.01	9 (69.2)	7 (36.8)	0.07	9 (69.2)	5 (31.3)	0.04
MR time from onset (hour)	25.5 [30.4]	53.1 [41.7]	0.03	31.2 [34.9]	41.3 [37.2]	0.45	18.3 [17.6]	55.5 [44.4]	0.009
Hypertension	16 (80.0)	8 (53.3)	0.09	10 (76.9)	12 (63.2)	0.41	9 (69.2)	10 (62.5)	0.71
Diabetes	7 (35.0)	6 (40.0)	0.76	3 (23.1)	8 (42.1)	0.27	5 (38.5)	6 (37.5)	0.96
Hyperlipidemia	9 (45.0)	6 (40.0)	0.77	5 (38.5)	7 (36.8)	0.93	6 (46.2)	8 (50.0)	0.84
Smoking	7 (35.0)	9 (60.0)	0.14	5 (38.5)	9 (47.4)	0.62	4 (30.8)	10 (62.5)	0.09
Atrial fibrillation	6 (30.0)	2 (13.3)	0.25	4 (30.8)	4 (21.1)	0.53	5 (38.5)	1 (6.2)	0.03
**Baseline VFS**	-21.9 [7.7]	-27.7 [5.0]	0.02	-22.0 [8.5]	-29.6 [1.6]	<0.001	-24.6 [9.0]	-29.5 [4.5]	0.07
**Lesion volume**	20.8 [14.4]	34.0 [23.8]	0.05	19.6 [14.2]	34.5 [20.9]	0.03	20.6 [13.1]	31.6 [24.2]	0.15
**Regional extent of ischemic lesion (%)**									
Lingual	21.1 [21.5]	49.8 [27.0]	0.001	16.1 [14.6]	50.3 [24.7]	<0.001	18.6 [24.3]	41.3 [27.7]	0.03
Calcarine	10.6 [15.2]	44.1 [26.2]	<0.001	9.8 [14.2]	36.4 [29.3]	0.005	10.6 [15.5]	43.3 [26.4]	<0.001
Fusiform	19.5 [21.3]	24.0 [21.9]	0.55	18.4 [22.9]	26.9 [19.9]	0.27	19.3 [21.7]	21.5 [21.7]	0.78
Cuneus	7.0 [15.1]	25.5 [26.4]	0.01	9.1 [18.2]	18.7 [26.0]	0.26	6.4 [11.3]	27.5 [26.7]	0.01
Inferior occipital	14.3 [21.1]	13.1 [13.7]	0.85	15.4 [21.6]	14.8 [16.5]	0.93	15.4 [21.7]	12.6 [14.2]	0.69
Parahippocampal	7.6 [11.2]	13.7 [16.0]	0.19	5.5 [8.6]	15.1 [15.6]	0.05	7.5 [12.5]	12.6 [15.7]	0.35
Hippocampus	5.1 [10.5]	12.6 [18.6]	0.14	2.5 [4.3]	13.6 [18.3]	0.04	5.7 [12.6]	11.4 [18.0]	0.34
Superior occipital	4.6 [12.2]	10.7 [17.2]	0.23	6.7 [14.8]	8.3 [15.9]	0.78	6.6 [14.8]	10.3 [16.7]	0.54
Middle occipital	6.0 [18.4]	5.5 [13.2]	0.93	9.0 [22.5]	4.5 [11.8]	0.46	9.2 [22.4]	5.1 [12.8]	0.54

Results are expressed in number and (% column) or mean [SD]. VFD: visual field defect, VFS: visual field score

The relative changes of visual field scores were significantly associated with left hemisphere infarct, baseline visual field score and rEIL of the lingual and calcarine cortices for hemi-, upper- and lower-VFDs (p < 0.05). The time from stroke onset to MRI scan was significantly associated with improvements of hemi- and lower-VFDs (p < 0.05). The presence of atrial fibrillation and rEIL of cuneus cortex were significantly associated with the relative changes of visual field scores in lower-VFD only (p < 0.05; Table 2).

**Table 2:** Correlation between predictive factors and relative changes of VFS (%VFS).

	Hemi-VFD		Upper-VFD		Lower-VFD	
	*p*-value	*r*	*p*-value	*R*	*p*-value	*r*
**Clinical variables**						
Age	0.31	0.18	0.24	0.22	0.27	0.21
Sex	0.81	-0.04	0.68	-0.08	0.95	-0.04
Left PCA infarction	0.008	0.44	0.03	0.38	0.02	0.42
MR time from onset (hour)	0.01	-0.42	0.19	-0.24	0.002	-0.54
Hypertension	0.52	0.11	0.67	0.08	0.94	0.02
Diabetes	0.58	-0.10	0.30	-0.19	0.91	0.02
Hyperlipidemia	0.89	-0.02	0.60	0.10	0.57	-0.11
Smoking	0.44	-0.13	0.54	-0.11	0.21	-0.24
Atrial fibrillation	0.13	0.26	0.44	0.14	0.045	0.37
**Baseline VFS**	0.002	0.51	<0.001	0.68	0.03	0.41
**Lesion volume**	0.13	-0.26	0.07	-0.32	0.29	-0.20
**Regional extent of ischemic lesion**						
Lingual	0.001	-0.52	<0.001	-0.62	0.049	-0.37
Calcarine	<0.001	-0.54	0.02	-0.41	0.002	-0.55
Fusiform	0.83	-0.04	0.34	-0.17	0.71	0.07
Cuneus	0.07	-0.31	0.44	-0.14	0.03	-0.41
Inferior occipital	0.34	0.17	0.90	0.02	0.14	0.28
Parahippocampal	0.13	-0.26	0.06	-0.34	0.38	-0.17
Hippocampus	0.09	-0.29	0.07	-0.32	0.31	-0.19
Superior occipital	0.62	-0.09	0.95	0.01	0.50	-0.13
Middle occipital	0.40	0.15	0.29	0.19	0.56	0.11

VFD = visual field defect; VFS = visual field score; %VFS = (follow-up VFS—baseline VFS) / |baseline VFS |.

### rEIL and visual field prognosis

**Fig 3:**
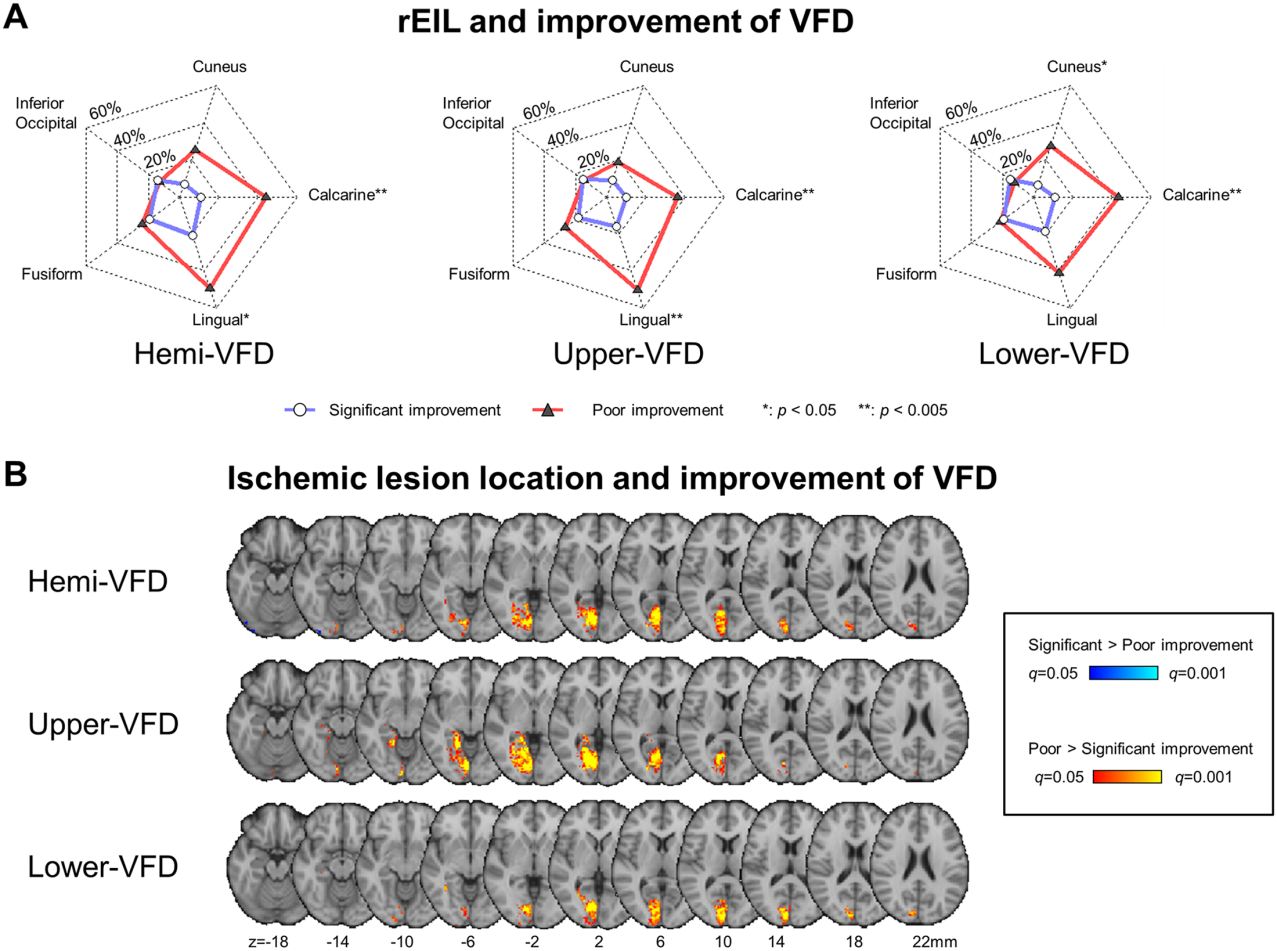
rEIL and the improvement of VFD. (A) Quantitatively represented location of the gyrus involved. White-filled circles with red-colored solid lines indicate significant improvement group (i.e., %VFS > 20%) and gray-colored triangles with blue-colored solid lines indicate poor improvement group (i.e., %VFS < 20%). (B) Differences in lesion patterns between significant and poor improvement groups were analyzed using the two-sample test for Bernoulli distribution False discovery rate correction based significance was determined at q < 0.05 (z = 2.825 for hemi-VFD; z = 2.813 for upper-VFD; and z = 2.914 for lower-VFD). Ipsilesional and contralesional hemispheres were presented in left and right side of brain, respectively.

**Fig 4:**
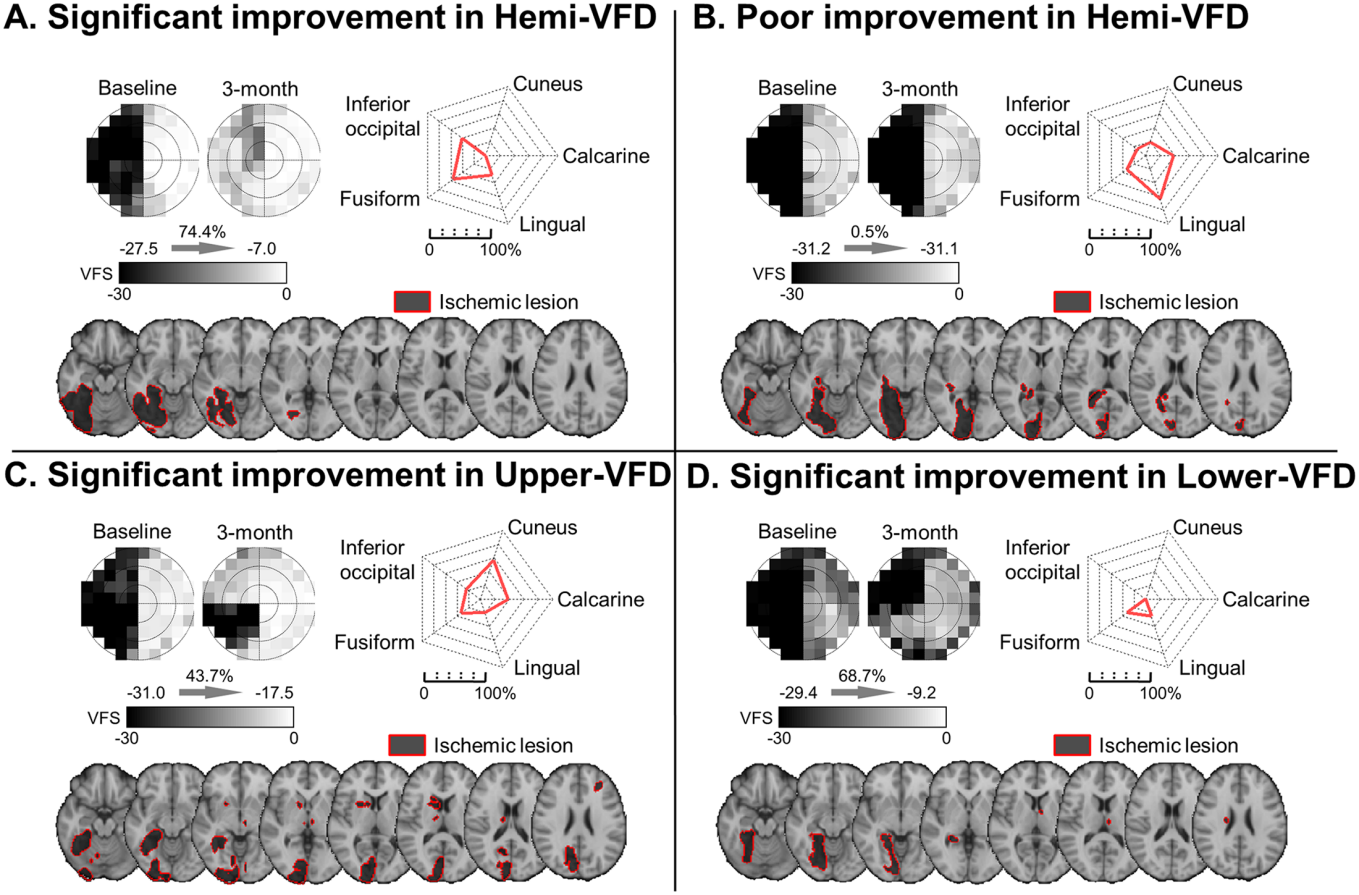
Representative cases of VFD pattern, rEIL and lesion location. (A) Significant improvement in hemi-VFD, (B) Poor improvement in hemi-VFD, (C) Significant improvement in upper-VFD, (D) Significant improvement in lower-VFD. (Top-left in A-D) Gray scaled visual field was presented. (Top-right in A-D) Red-colored solid lines indicate quantified lesion location by regional extent of ischemic lesion. (Bottom in A-D) Lesion area was superimposed on standard MNI T1 scan. The border line and area of lesion were shown with red-solid line and dark area.

High rEIL was found in the lingual (33.4 ± 4.7%), calcarine (24.9 ± 4.5%), fusiform cortex (21.4 ± 3.7%), and cuneus (14.9 ± 3.8%). A significant improvement for hemi-VFD demonstrated less rEIL in the calcarine, lingual and cuneus. In addition to calcarine and lingual cortex, significant improvements were associated with less involvement of the lingual cortex and hippocampus in upper-VFD, and cuneus in lower-VFD (Table 1, Fig 3). Fig 4 shows the representative patients with their VFD improvement and lesion location.

### Factors associated with visual field improvement by univariate analysis

**Fig 5:**
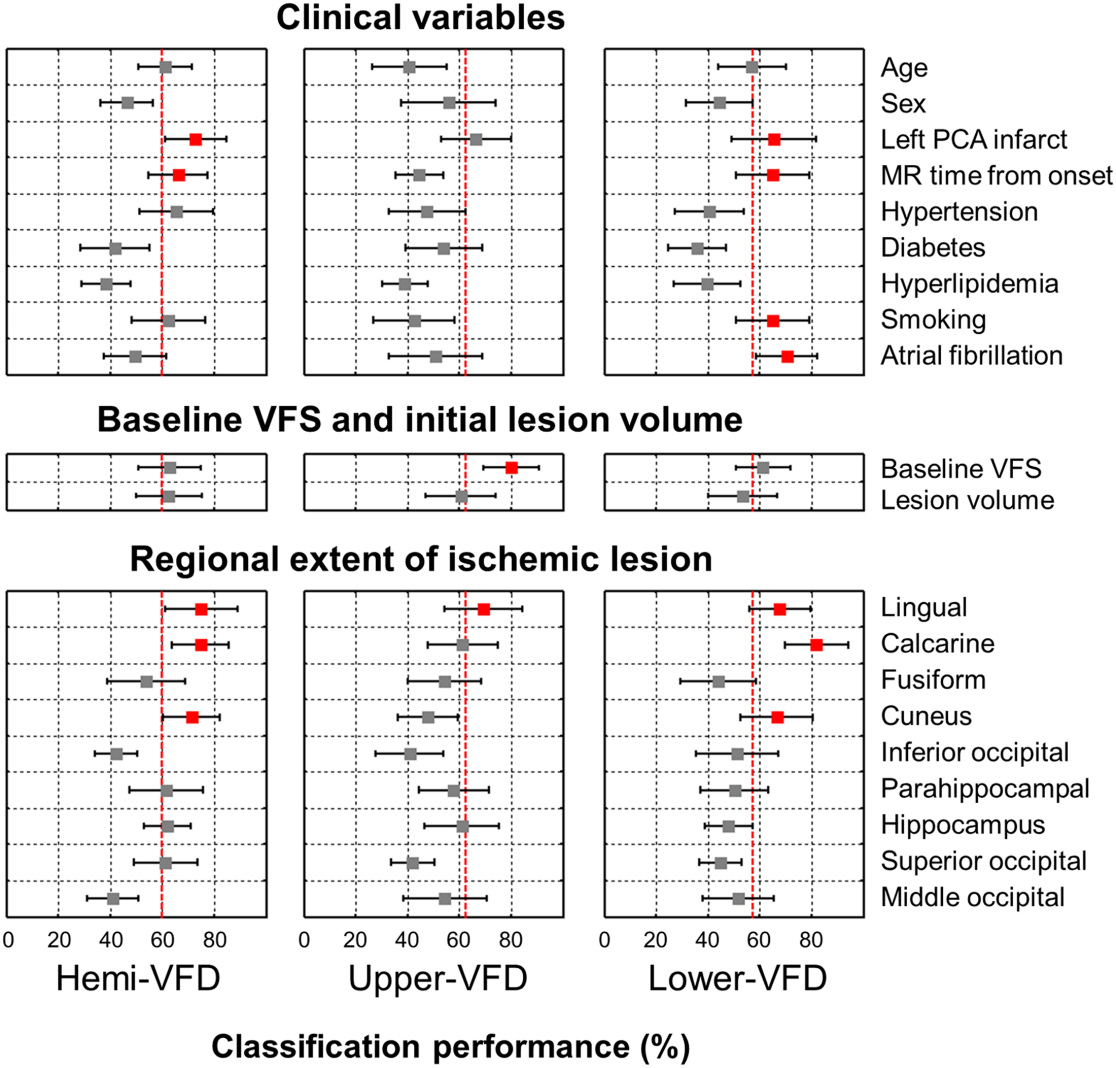
Performance of each variable from the classification test. Mean and standard deviation across 100 times of classification test were plotted. Significantly greater performance than random chance was filled with red color (*p* < 0.001). Dotted red-line represents the random chance. Random chance = (greater number of subjects among significant and poor improvement group) / total number of subjects for each hemi-VFD, upper-VFD and lower-VFD, respectively.

According to the classification tests based on single variables, the variables demonstrating the highest performance for predicting significant hemi-VFD improvement was rEIL of the lingual cortex (classification performance predicting the prognosis: 75.0 ± 13.9%; *p* < 0.001), calcarine cortex (74.7 ± 10.9%, *p* < 0.001), cuneus (71.3 ± 10.8%, *p* < 0.001) left PCA infarct (72.8 ± 11.8%, p < 0.001), MR time from onset (66.1 ± 11.2%, p < 0.001)and hypertension (65.6 ± 14.2%, *p* = 0.01) comparing to the random chance of 60%. Significant upper-VFD improvement was well predicted by the baseline visual field score (80.0 ± 10.8%, *p* < 0.001) and rEIL of lingual cortex (69.5 ± 15.0%, *p* < 0.001) comparing to the random chance of 62.5%. Significant lower-VFD improvement was well predicted by rEIL of the calcarine cortex (81.9±12.2%, *p*<0.001), lingual cortex (67.7 ± 11.8%, *p* < 0.001), cuneus (66.7 ± 13.9%, *p* < 0.001), atrial fibrillation (70.4 ± 11.9%, *p* < 0.001), left PCA infarct (65.4 ±16.4%, p < 0.001), MR time from onset (65.0 ± 14.0%, p < 0.001), smoking (65.0 ± 14.1%, *p*<0.001) and baseline visual field score (61.3±10.6%, *p* = 0.02) comparing to the random chance of 57.1% (Fig 5).

### Subgroups and combination of subgroups for visual field improvement prediction

**Fig 6:**
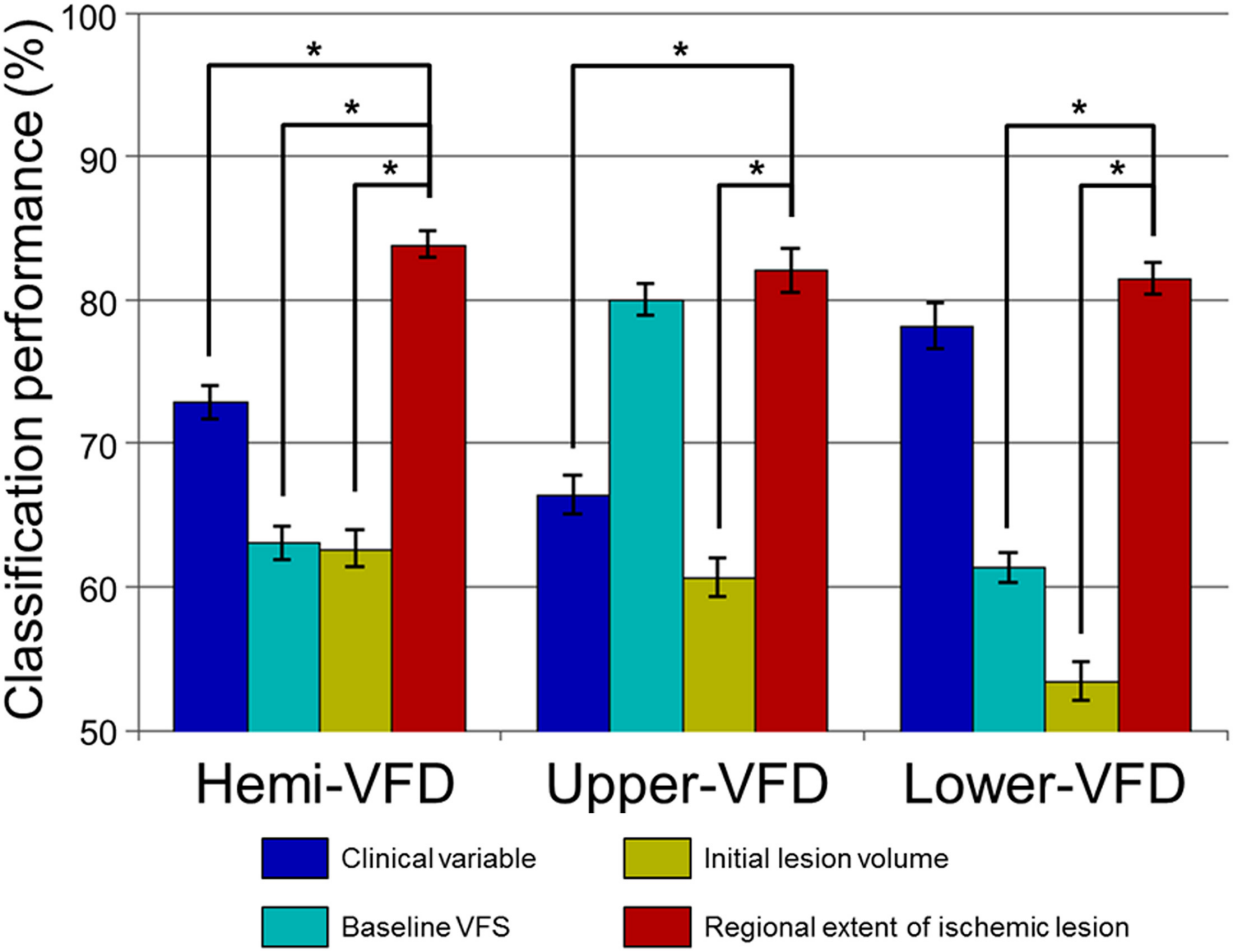
Maximum classification performances predicting the improvement of VFD. Mean and standard error of maximum classification performance were plotted for each subgroup features. Asterisk (*) represents statistically significant differences from paired t-test across 100 times of classification test (*p* < 0.05).

The prediction performance of the visual field improvement was highest using the rEIL (hemi-VFD: 83.8±9.5%, upper-VFD: 82.0 ± 15.0%, and lower-VFD: 81.4 ± 11.0%) compared with clinical variables (hemi-VFD: 72.8 ± 11.8%, *p*<0.001; upper-VFD: 66.4 ± 13.5%, *p* < 0.001; and lower-VFD: 78.1 ± 16.0%, no significance), baseline VFD (hemi-VFD: 63.0 ± 11.9%, *p* < 0.001; and lower-VFD: 61.3 ± 10.6%, *p* < 0.001), or initial ischemic lesion volume (hemi-VFD: 62.6 ± 12.7%, *p* < 0.001; upper-VFD: 60.6 ± 13.5%, *p* < 0.001; and lower-VFD: 53.4 ± 13.4%, *p* < 0.001; Fig 6). The baseline VFD failed to show a significant difference with rEIL for predicting the significant improvement of upper-VFD.

**Fig 7:**
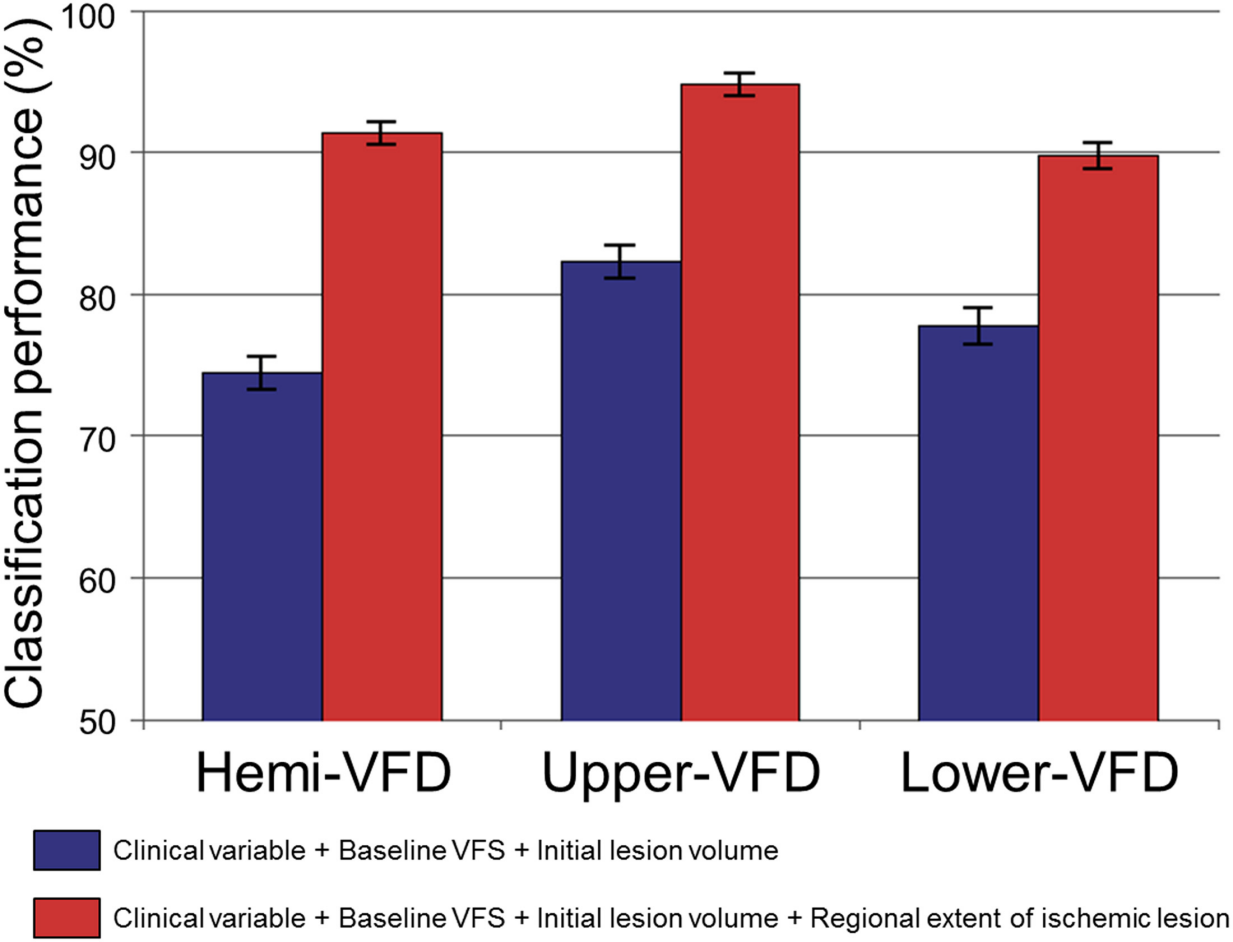
The effect of adding rEIL to other variables on predicting VFD improvement. Mean and standard error of maximum classification performance were plotted without (blue-colored bar plot) and with (red-colored bar plot) including the quantified lesion location features (e.g., regional extent of ischemic lesion).

Adding rEIL information to the prediction model including clinical variables, baseline VFD, and initial lesion volume enhanced the performance for predicting the significant improvement of VFD; hemi-VFD: from 74.4 ± 11.6% to 91.3 ± 7.7% (*p* < 0.001), upper-VFD: from 82.3 ± 11.5% to 94.8 ± 8.2% (*p* < 0.001) and lower-VFD: from 77.7 ± 12.9% to 89.7 ± 9.5% (*p* < 0.001; Fig 7).

## Discussion

In the present study, we demonstrated the usefulness of a quantitative analysis of lesion location based on a standard atlas for predicting the significant VFD improvement after PCA infarction. Less involvement of the calcarine, lingual, and cuneus cortices was associated with significant VFD improvement. A quantified lesion location showed a better performance for predicting the significant improvement than ordinary clinical variables or a combination of those variables. In addition, adding rEIL information to the classification test enhanced its performance for predicting the significant VFD improvement with ordinarily used clinical variables.

Various clinical variables including age, previous stroke history, and the initial severity after stroke well correlate with improvement of various symptoms after stroke. Previously, one of the clinical factors associated with significant improvement of VFD was the time from the injury to evaluation.[14] In the present study, we have comprehensively compared the performance of each clinical variable predicting the improvement of VFD. Left-sided PCA infarction and short interval between stroke onset and MRI were associated with significant hemi-VFD improvement. This finding corresponds well with the previous studies showing that the left-sided hemispheric lesions improve better, [15,16] and that shorter interval between stroke onset and hospital admission is associated with significant improvement.[14]

Considering the stroke location, our current results are consistent with those of a previous study demonstrating that the involvement of the striate cortex and lesion expansion to the occipital pole was associated with poor VFD recovery [17]. In the present study, not the total lesion volume, but the rEIL of the lingual cortex demonstrates a high performance for predicting the improvement of hemi-, upper-, and lower-VFD. Visual field recovery is likely to be mediated by the immediate surrounding residual neuronal elements to the infarcted visual cortex, which plays a major role in receptive field plasticity [18] Therefore, brain damage of a specific region, may be more important in vision restoration than the overall ischemic lesion volume.

However, only a few studies have focused on lesion location-based prediction for the prognosis of stroke. A multivariate linear regression model with lesion-weighted CT perfusion data has accurately predicted the improvement of motor and language function after stroke [5,19]. Compared to CT, MRI provides a more detailed structure of the brain and may therefore be more appropriate for predicting lesion- based prognosis [20,21]. Recently, the outcome and recovery of language function was appropriately predicted using SVM with lesion-location data from MRI [8]. The results were consistent with our current findings in that atlas-based lesion data showed additive value to enhance the power of variance for predicting prognosis.

Interestingly, the factors showing the best predictive performance slightly differed between upper- and lower-VFDs. Only the baseline visual field score and rEIL of lingual cortex demonstrated a significantly higher performance predicting the prognosis of upper-VFD. The lingual cortex is supplied by the calcarine artery and is located close to the PCA making them more frequently involved and more likely to be located at the infarct core [22]. Therefore, the initial severity may be the major deterministic factor of the prognosis of upper-VFD. However, still rEIL significantly increased the classification performance when it was added to other variables (Fig 7). In contrast, infarct volume or baseline visual field score was not found to be associated with the prognosis of lower-VFD (Fig 5). Instead, the rEILs of the calcarine, lingual cortex and cuneus were a good prognostic indicator. These results imply that the specific lesion location itself plays a relatively more important role in the prognosis of lower-VFD. Consistent with our results, previous studies have demonstrated that the recovery of lower-VFD is better than that of upper-VFD after ischemic stroke and that the preservation of the cuneus cortex is associated with restoration of lower-VFD [23].

Still there may be some debates in defining 20% improvement from the baseline visual field score as a significant VFD improvement. Patients with mild VFD may not reach the 20% improvement due to ceiling effect. However, there was not any patient who was classified as non-significant improvement due to the ceiling effect, and even the mildest patient in our sample demonstrated a VFD enough to improve more than 20% before reaching the normal score. Furthermore, the analysis using the absolute difference in the visual field score from baseline to follow-up (ΔVFS) did not show significant major difference from the analysis to that of using percent improvement from baseline (%VFS). From the previous studies, the size of the relative vision was used for evaluating the outcome, [24] [25] and much smaller improvement rates were considered clinically meaningful. [26] In the present study we used a median split to dichotomize the patients and perform a classification test using SVM. This may be arbitrary and lead to a bias. However, the correlation analysis between variables and the relative change of visual field score demonstrated similar results (Table 2). Further discussion may be needed in the method and cut-off value defining significant improvement in VFD after stroke.

There are some additional noteworthy limitations to this study. First, sample size was small. However, we performed a test-retest classification to ensure the reliability of our results. Second, recanalization of the PCA was not checked, which may influence the functional recovery. Third, while this lesion location-based prognosis prediction model accurately predicted the improvement of VFD, other stroke symptoms (*e*.*g*., motor weakness and aphasia) should also be evaluated in this model to expand its clinical application. Finally, though most VFD following stroke has largely been attributed to cortical lesions, infarct at the different visual areas such as optic radiation fibers was not considered in the present study. Further studies using diffusion tensor image or functional connectivity MRI to predict the improvement of VFD may be needed. [27]

## Conclusions

In conclusion, the lesion location based on rEIL of each individual cortex—which is easily applicable in the clinical practice—accurately predicts the improvement of VFD. Adding rEIL data to clinical variables improves the performance of a prediction model for VFD after PCA infarction. Furthermore, because the predictive factors differ between upper and lower visual field, the mechanism of restoration of upper- and lower-VFDs may also be different.

## Supporting Information

S1 Data Raw data. (XLSX)
